# The Role of Geranylgeraniol in Managing Bisphosphonate-Related Osteonecrosis of the Jaw

**DOI:** 10.3389/fphar.2022.878556

**Published:** 2022-05-04

**Authors:** Kok-Yong Chin, Sophia Ogechi Ekeuku, Anne Trias

**Affiliations:** ^1^ Department of Pharmacology, Faculty of Medicine, Universiti Kebangsaan Malaysia, Cheras, Malaysia; ^2^ American River Nutrition, Hadley, MA, United States

**Keywords:** angiogenesis, bone, gingival fibroblast, osteoblasts, osteoclasts

## Abstract

Medication-related osteonecrosis of the jaw (ONJ) is a rare but significant adverse side effect of antiresorptive drugs. Bisphosphonate-related ONJ (BRONJ) is the most prevalent condition due to the extensive use of the drug in cancer and osteoporosis treatment. Nitrogen-containing bisphosphonates suppress osteoclastic resorption by inhibiting farnesyl pyrophosphate synthase in the mevalonate pathway, leading to deficiency of the substrate for GTPase prenylation. The bone remodelling process is uncoupled, subsequently impairing bone healing and causing ONJ. Targeted administration of geranylgeraniol (GGOH) represents a promising approach to mitigate BRONJ because GGOH is a substrate for GTPase prenylation. In the current review, the *in vitro* effects of GGOH on osteoclasts, osteoblasts and other related cells of the jaw are summarised. We also present and appraise the current *in vivo* evidence of GGOH in managing BRONJ in animal models. Lastly, several considerations of using GGOH in the clinical management of BRONJ are highlighted. As a conclusion, GGOH is a promising topical agent to manage BRONJ, pending more research on an effective delivery system and validation from a clinical trial.

## Introduction

Osteonecrosis of the jaw (ONJ) is a rare adverse reaction towards medications that suppress bone resorption or angiogenesis. It is prominently associated with prolonged high-dose bisphosphonate administration, usually indicated for the treatment of cancer rather than osteoporosis. Other medications that have been associated with ONJ include denosumab (an antiresorptive), bevacizumab (an antiangiogenic) and tyrosine kinase inhibitors. Collectively, ONJ caused by these medications is called medication-related ONJ (MRONJ). ONJ is characterised by impaired bone healing of the craniofacial region (Chang et al., 2018; Kawahara et al., 2021). Clinically, this condition is diagnosed based on 1) exposed maxillofacial bone that is palpable through intraoral or extraoral fistula and does not heal within 8 weeks; 2) prior or current exposure to antiresorptive or antiangiogenic agents; 3) the absence of radiation therapy or apparent metastatic disease at the maxillofacial region (Ruggiero et al., 2014; Japanese Allied Committee on Osteonecrosis of the Jaw et al., 2017).

The unique anatomy and environment of the jaw predispose it to ONJ. The oral cavity serves as a habitat to a host of microorganisms, which can cause infection and inflammation directly to the jaw bone through the gap between the teeth and the oral epithelium or root canal, or indirectly through periodontal/periapical diseases and tooth decay, or any dental treatments that injure the oral mucosa (Japanese Allied Committee on Osteonecrosis of the Jaw et al., 2017). The pathogenesis of MRONJ remains elusive. Exposure to antiresorptive and antiangiogenic agents does not constitute an absolute cause because most patients do not develop MRONJ. The prevailing hypothesis is that MRONJ is a multifactorial disease (Kim et al., 2021). The combination of infection and inflammation caused by bone trauma (e.g. tooth extraction), suppressed bone remodelling by antiresorptive agents, and inhibition of new blood vessel formation by antiangiogenic agents contribute to the disease (Aghaloo et al., 2015). In addition, oral bisphosphonates can cause soft tissue toxicity which harms the mucosal layer (Psimma et al., 2021). However, the prevalence of MRONJ caused by denosumab, which lacks soft tissue toxicity, is as high as bisphosphonates (Stopeck et al., 2016; Srivastava et al., 2021), indicating it does not play a central role in disease development. In line with the pathogenesis, dental infection, poor oral hygiene, and bone-invasive dental treatment such as tooth extractions, dental implants and dentures are risk factors of MRONJ (Shibahara, 2019).

A meta-analysis of 12 observational studies involving patients with cancer receiving sequential bisphosphonate/bisphosphonate-denosumab therapy reported that the weighted pooled prevalence of MRONJ associated with sequential pamidronate-zoledronate therapy is 19% (95% confidence interval (CI) 10–27%), bisphosphonate-denosumab is 13% (95%CI 3–22%) and ibandronate-zoledronate is 10% (95% CI 3–22%). MRONJ prevalence for bisphosphonates per se was 5% (95% CI 0–9%) and denosumab per se is 4% (95% CI 3–5%) (Srivastava et al., 2021). However, studies included in this meta-analysis were highly heterogeneous with a wide range of MRONJ onset. A comparative study has highlighted that MRONJ occurs earlier in patients receiving sequential bisphosphonate-denosumab, especially during the first year after switching, compared to the bisphosphonate group (Loyson et al., 2018). The analysis of three large prospective trials of advanced breast cancer reported that MRONJ risk increases in patients receiving bevacizumab with bisphosphonate exposure (incidence: 0.9–2.4) compared to those without (incidence: 0–0.2%) (Guarneri et al., 2010).

Two major mitigation approaches of MRONJ currently are preventive premedication dental evaluation and drug holiday (Kim et al., 2021). A premedication dental evaluation involves baseline oral health assessment, scheduled follow up, oral care instructions, antiseptic rinses, preventive periodontal treatment, extraction of non-restorable teeth and denture adjustment. A meta-analysis of six studies reported that these measures reduced MRONJ incidence among cancer patients by 77.3% (95% CI 47.4–90.2%) (Karna et al., 2018). For patients at high risk for ONJ, withholding antiresorptive therapy before and following extensive oral surgery until the complete healing of the surgical site could be considered (Wan et al., 2020). However, for patients with serious diseases such as cancer and immunosuppression, cessation of treatment to reverse MRONJ is not recommended due to the lack of evidence on its benefits and concerns on disease relapse (Kawahara et al., 2021). The optimal treatment for MRONJ has not been established. In the early stages, surgical debridement and coverage, hyperbaric oxygen, and low-intensity laser therapy are effective. In severe cases, a segmental osteotomy is recommended (Rasmusson and Abtahi, 2014). The quest for an optimal strategy to manage MRONJ is ongoing, given the lack of effective options.

**FIGURE 1:**
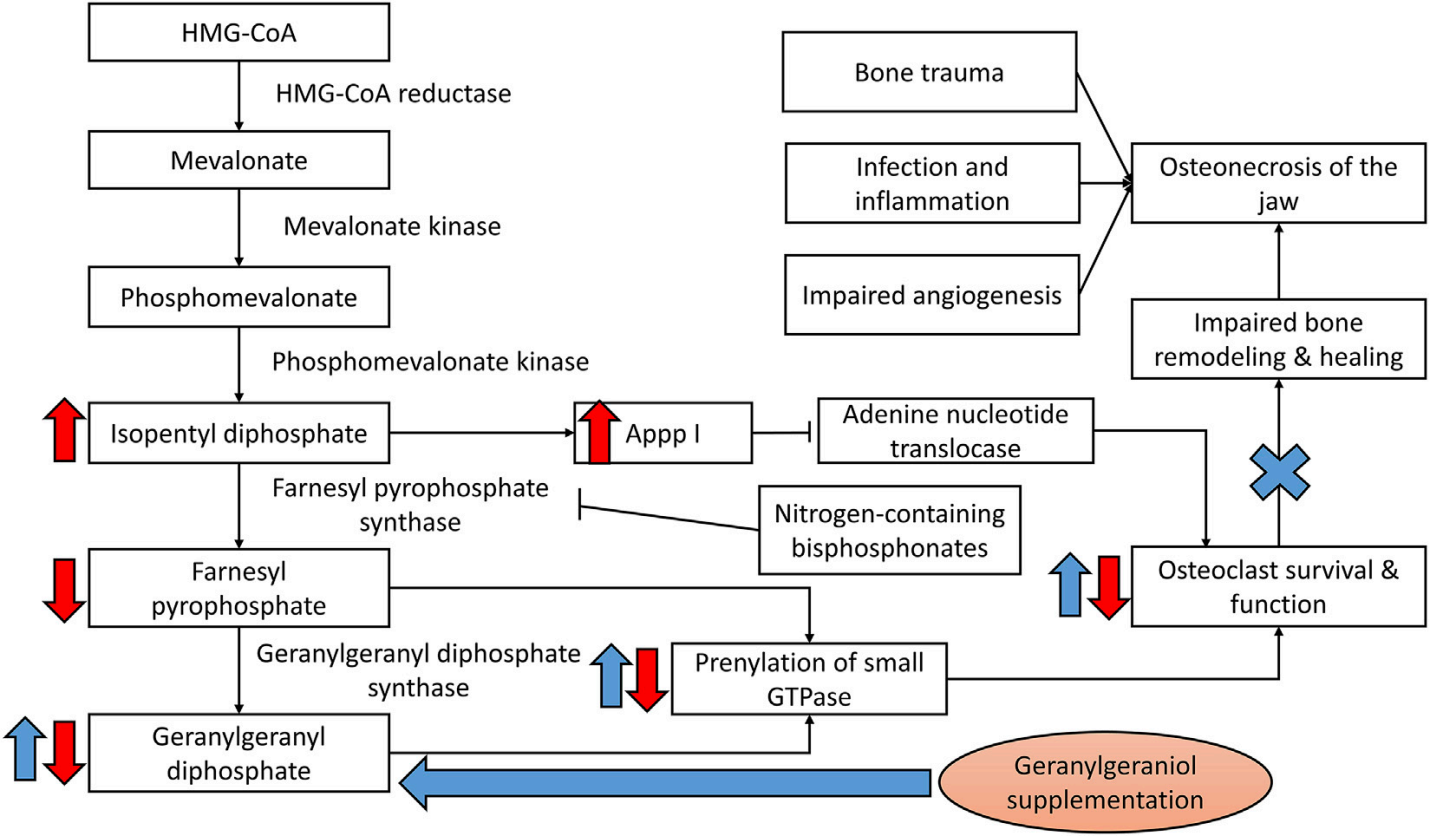
Bisphosphonates in the development of ONJ. Geranylgeraniol could replenish the substrate for prenylation of GTPase, thereby reactivating the bone remodelling cycle and healing process to mitigate osteonecrosis of the jaw induced by bisphosphonates. Notes: Red arrows indicate the effects of bisphosphonates; blue arrows indicate the potential effects of geranylgeraniol supplementation.

Since bisphosphonates pose a higher risk of ONJ compared to other medications and are used prevalently for osteoporosis and cancers, they are the focus of the current review. Nitrogen-containing bisphosphonates act by inhibiting farnesyl pyrophosphate synthase of the mevalonate pathway, thereby blocking the generation of substrate for protein prenylation in osteoclasts. This event will suppress differentiation, survival and function of the osteoclasts, and ultimately interferes with bone resorption rate (Hasan et al., 2018; Cremers et al., 2019). Nitrogen-containing bisphosphonates could also induce osteoclast apoptosis by inhibiting adenine nucleotide translocase due to the formation of ATP analogues as a result of mevalonate pathway suppression (Mönkkönen et al., 2006). Since bone remodelling is a coupled process, bone healing following trauma is also suppressed, especially in the presence of inflammation and infection (Loi et al., 2016). Thus, the revival of normal bone remodelling at the jawbone represents an avenue of intervention (Figure 1). Apart from that, bisphosphonates are toxic to gingival fibroblasts, blood vessel epithelial cells and osteoblasts important in mucosal and bone healing (Ziebart et al., 2011; Hagelauer et al., 2015).

Many previous studies have utilised geranylgeraniol (GGOH), a diterpene alcohol or a polyprenol, to antagonise the effects of nitrogen-containing bisphosphonates. GGOH has been discovered as a wax ester from olives, annatto, sunflower, hemp and oil palm (Reiter and Lorbeer, 2001; Montserrat-de la Paz et al., 2014; Lucci et al., 2015; Silva et al., 2015). Being a central isoprenoid in the mevalonate pathway, GGOH is not only a substrate for the synthesis of menatetrenone/menaquinone-4 (MK-4) and a building block for CoQ10 (Shirakawa et al., 2018), but also acts as an important intermediate for the prenylation of proteins by GTPases (Shirakawa et al., 2018), providing the basis for its protective actions against bisphosphonate-related ONJ (BRONJ). Most recently, GGOH has been commercialized as a dietary supplement in the form of an extract from annatto, and is generally recognized as safe (GRAS) (Miyawaki et al., 2020), opening the doors to potential clinical trials with the endogenous nutrient.

This review aims to summarise the evidence of GGOH as an agent to prevent BRONJ. The biological effects of GGOH on osteoclasts, osteoblasts and other cells alone or in the presence of bisphosphonates are discussed. The *in vivo* evidence of GGOH in preventing BRONJ are also appraised and presented. We hope this review will provide the impetus for interested researchers to further explore the effects of GGOH as an interventional agent against BRONJ, and present a platform from which to launch human trials to illuminate the compound’s benefits clinically.

### Cellular Effects of GGOH

To better understand the following content, the readers should know that osteoclasts originate from haematopoietic stem cells. The differentiation of these progenitors into functional, tartrate-resistant acid phosphate (TRAP)-positive multinucleated osteoclasts requires signals from osteoblasts from the mesenchymal lineage. *In vitro* experiments, osteoclasts are differentiated from murine spleen cells, murine or human macrophages cocultured with osteoblasts/stromal cells or osteoclastic differentiation stimulants. Osteoclasts initiate the bone remodelling cycle by degrading the bone matrix. Osteoblasts could be isolated from the long bones of animals or are available as commercial cell lines (e.g. MC3T3-E1 murine pre-osteoblasts) cultured with osteogenic medium (Jolly et al., 2018; Sieberath et al., 2020; Jolly et al., 2021). Osteoblasts rebuild bone by synthesising the bone matrix and mineralising it.

### Effects of GGOH Alone on Osteoclasts

Earlier studies recognised that GGOH is the isoprenoid side chain of MK-4, one of the nine isoforms of vitamin K2 and might contribute to the biological activities of MK-4 (Ho et al., 2018). MK-4 displays the unique ability to suppress the formation of osteoclasts from bone marrow macrophages more effectively than other vitamin K isoforms. This property is not shared by vitamin K1 (Wu et al., 2015). Hara et al. reported that GGOH and MK-4 shares a similar ability to suppress TRAP-positive cell formation in primary murine spleen cultured with TMS stromal cells induced by 1,25-dihydroxyvitamin D3 (1,25(OH)D3), a stimulator of osteoclastogenesis. This effect is not shared by vitamin K1, phytol and other variations of similar molecules (geraniol, farnesol, generanylfarnesol, farnesylfarnesol and geranylgeranyl-farnesol) (Hara et al., 1995). Taira et al. demonstrated similar findings using human peripheral monocytes induced by RANKL and MCSF, and bone resorption activities of TRAP-positive cells *in vitro* was inhibited by MK-4 and GGOH (Taira et al., 2003). Hiruma et al. reported that 1,25(OH)D3 upregulates prostaglandin E2 and cyclooxygenase-2 expressions to stimulate osteoclast formation. In the same study, they found that GGOH is effective in suppressing TRAP-positive cells formation in co-culture of murine spleen cells and TMS bone marrow stromal cells induced by prostaglandin E2, while MK-4 is effective in suppressing a similar process induced by 1,25(OH)D3. These results indicate that GGOH act on pathways downstream of prostaglandin E2 signalling, while MK-4 acts on 1,25(OH)D3 signalling to inhibit osteoclast formation. As evidence, GGOH also reduces prostaglandin E2 production, cyclooxygenase-2 and RANKL expression in the co-culture (Hiruma et al., 2004).

Others have demonstrated the opposite results by showing that GGOH promotes osteoclast formation and bone resorption. Using murine foetal metatarsal explant culture, van Beek et al. reported that the effects of GGOH are dose-dependent, in which osteoclastic resorption is promoted at low doses (50–100 μM) but suppressed at high dose (500 μM) probably due to its cellular toxicity effects (van beek et al., 1999). Similar toxicity effects have been replicated by Fliefel et al., whereby low-dose GGOH (10–40 μM) promoted osteoclast viability but the inverse occurs at a high dose (80 μM) (Fliefel et al., 2019). Regarding the divergent actions of GGOH in different culture models, van Beek demonstrated that the effects of GGOH on osteoclasts are partially mediated by the retinoic acid receptor (van Beek et al., 2006), which is also the target of 1,25(OH)D3. In the foetal metatarsal explant model used by van Beek et al., which the authors claimed to mimic the natural bone environment better, GGOH promotes osteoclastic resorption (van Beek et al., 2006). In other *in vitro* osteoclast models, GGOH might compete with 1,25(OH)D3 to bind with retinoic acid receptor, a more effective osteoclastogenesis stimulant, leading to reduction of osteoclastic resorption (Table 1).

**TABLE 1:** The effects of GGOH on osteoclasts alone.

Authors (years)	Study design	Major findings
Hara et al. (1995)	Primary murine spleen cells cultured with TMS stromal cells in the presence of 1,25(OH)D	↓ TRAP activity, TRAP + cells by GGOH (10^−6^ to 10^−5^ M) and MK-4
		Vitamin K1, phytol, geraniol, farnesol, generanylfarnesol, farnesylfarnesol and geranylgeranyl-farnesol - no similar effects
Benford et al. (1999)	J774 macrophages incubated with bisphosphonates with or without GGOH.	↓ caspase-3-like activity induced by aminobisphosphonates by GGOH (10^−4^ M)
van Beek et al. (1999)	Murine foetal metatarsals cultured with different additives for three days. Resorption was calculated as calcium-45 in the medium/calcium-45 in the bone	Low dose (5 × 10^−5^–1 × 10^−4^ M) ↑ osteoclastic resorption; High dose (5 × 10^−4^ M) ↓ osteoclastic resorption
Taira et al. (2003)	Human peripheral monocytes cultured with RANKL, MCSF and vitamin K/GGOH	↓ TRAP + cells and resorption activity by MK-4 and GGOH (10^−6^–10^−5^ M)
Hiruma et al. (2004)	Murine spleen cells co-cultured with TMS-12 bone marrow stromal cells in the presence of soluble RANKL, 1,25(OH)D3 and PGE2	↓ TRAP + cells induced by 1,25(OH)D3 by MK-4 (5 × 10^−6^ M)
		↓ TRAP + cells induced by PGE2 by GGOH (5 × 10^−6^ M)
van Beek et al. (2006)	Foetal metatarsals labelled with calcium-45	↑ osteoclastic resorption by RA, GGOH (10^−6^–10^−4^ M), GGA (1 × 10^−6^ M–5 × 10^−5^ M) and GGPP (10^−6^–10^−4^ M)
Nagaoka et al. (2015)	Bone marrow macrophages stimulated with RANKL, and MCSF in the presence or absence of zoledronate (0–10^−5^ M) with or without GGOH for 4 days	GGOH (3 × 10^−6^ M) and GGPP (3 × 10^−6^ M) ↑ TRAP + cells with >11 nuclei, expression of osteoclast markers (TRAP, DC-STAMP, OC-STAMP, cathepsin K, calcitonin receptor)

Abbreviation↓, decrease; ↑, increase; +, positive; 1,25(OH)D, 1,25-dihydroxyvitamin D3; DC-STAMP, dendrocyte expressed seven transmembrane protein; GGOH, geranylgeraniol; MCSF, macrophage colony-stimulating factor; MK-4, menatetrenone; RANKL, receptor activator of nuclear factor kappa-Β ligand; NFATc1, nuclear factor of activated T-cells, cytoplasmic 1; OC-STAMP, osteoclast stimulatory transmembrane protein; PGE2, prostaglandin E2; TNF, tumour necrosis factor; TRAP, tartrate-resistant acid phosphatase.

### Effects of GGOH on Osteoclasts in the Presence of Bisphosphonates

GGOH could antagonise the effects of alendronate in suppressing TRAP-positive cell formation and resorption area in murine bone marrow co-cultured with osteoblasts or primary leporine osteoclasts (Fisher et al., 1999). It can also prevent nuclear condensation and loss of actin rings in murine osteoclasts induced by risedronate or alendronate (Reszka et al., 1999; Hiruma et al., 2004). The suppression of Mst1 cleavage induced by risedronate or alendronate contributes to this event (Reszka et al., 1999). MK-4 did not share the property of GGOH in rescuing the actin ring of osteoclasts (Hiruma et al., 2004). However, GGOH cannot prevent apoptosis induced by non-nitrogen containing bisphosphonates, such as etidronate and clodronate (Reszka et al., 1999). Similarly, GGOH prevents suppression of osteoclast formation and bone resorption in murine foetal metatarsal explants induced by nitrogen-containing bisphosphonates, but not the non-nitrogen-containing bisphosphonates (van beek et al., 1999; Van Beek et al., 2002; Van Beek et al., 2003). These observations are expected because non-nitrogen containing bisphosphonates do not affect the cellular isoprenoid pool, which is the target of GGOH. However, GGOH only reverses pamidronate-mediated suppression of osteoclastic resorption partially, indicating that pamidronate may inhibit bone resorption through other mechanisms apart from the mevalonate pathway (Van Beek et al., 2003).

The reversal effects of GGOH are dependent on the dose of bisphosphonates. GGOH cannot reverse the effects of high-dose bisphosphonate resembling monthly or yearly administration (Fisher et al., 2013). Fisher et al. postulated that high-dose bisphosphonates deplete the cellular geranylgeranyl pyrophosphate pool beyond the level supplementation can reverse (Fisher et al., 2013). High-dose bisphosphonates also cause accumulation of proapoptotic ATP analogue, Appp I due to accumulation of isopentyl pyrophosphate and interaction with mitochondrial adenine nucleotide translocase (Mönkkönen et al., 2006). Since this step occurs before the action of farnesyl pyrophosphate synthase, it cannot be reversed by GGOH.

Other actions of GGOH include reversal of zoledronate-induced suppression of inward/outward chloride ion in the acidic environment (Ohgi et al., 2013), and reversal of zoledronate-induced suppression of NFATc1 and carbonyl anhydrase II expression (Nakagawa et al., 2015). In human peripheral mononuclear cells stimulated with RANKL and MCSF, GGOH downregulates tumour necrosis factor (TNF), chemokine ligand (CXCL) 9 and CXCL10, which are upregulated with zoledronic acid treatment (Zafar et al., 2020). TNF is a proinflammatory and antiangiogenic cytokine, which can upregulate CXCL9 and CXCL10 (Ohta et al., 2008), which are known to be antiangiogenic (Yates-Binder et al., 2012; Gao et al., 2017). In bone marrow macrophages stimulated with RANKL and MCSF, GGOH reactivates osteoclasts exposed to zoledronate by upregulating osteoclastic genes and proteins, such as TRAP, dendrocyte expressed seven transmembrane protein and osteoclast stimulatory transmembrane protein (Nagaoka et al., 2015). These events initiated by GGOH lead to increased protein expression of cathepsin K and calcitonin receptor, which are osteoclast differentiation markers (Nagaoka et al., 2015).

Overall, while the effects of GGOH alone on osteoclasts depends on the *in vitro* system used, it can negate the negative effects of nitrogen-containing bisphosphonates at a suitable dose range. Exceptions include high-dose bisphosphonates, which can activate non-mevalonate pathways that cause osteoclast apoptosis not restorable by GGOH (Table 2).

**TABLE 2:** The effects of GGOH on osteoclasts in the presence of bisphosphonates.

Authors (years)	Study design	Major findings
Fisher et al. (1999)	Murine bone marrow cultured with the MB1.8 osteoblasts in the presence of 1,25(OH)D3 and lovastatin/alendronate with or without GGOH. The experiment was repeated using rabbit osteoclasts from tibia/femur	GGOH (10^−5^ M) ↑ TRAP + cells and resorption area suppressed by lovastatin and alendronate
		GGOH prevents the activation of kinases in osteoclasts by lovastatin and alendronate
Reszka et al. (1999)	Murine bone marrow cells were cocultured with MB1.8 osteoblastic cells, after the formation of osteoclasts, osteoblasts were removed	↓ nuclear condensation and actin ring loss by GGOH (10^−5^ M) in risedronate and alendronate, not etidronate and clodronate-treated cells
		↓ cleavage of Mst1 by GGOH (10^−5^ M) in alendronate, risedronate and lovastatin-treated cells
van Beek et al. (1999)	Murine foetal metatarsals cultured with different additives for three days. Resorption was calculated as calcium-45 in the medium/calcium-45 in the bone	GGOH (5 × 10^−5^ M) ↑ osteoclastic resorption suppressed by ibandronate, risendronate, alendronate, olpadronate, not clodronate and etidronate
van Beek et al. (2002)	Metatarsal from 15-day-old mice cultured with different additives	GGOH (10^−4^ M) ↑ osteoclastic resorption suppressed by olpadronate, not clodronate
van Beek et al. (2003)	Foetal metatarsals labelled with calcium-45	↓ Efficacy of alendronate (EC_50_ increased from 6 × 10^−7^ M to 1 × 10^−4^ M) and risedronate (EC_50_ increased from 4 × 10^−6^ M to 1.5 × 10^−5^ M) in suppressing osteoclastic resorption
		↓ Efficacy of pamidronate partially (EC_50_ increased from 4 × 10^−6^ M to 1.5 × 10^−5^ M)
Hiruma et al. (2004)	Murine spleen cells co-cultured with TMS-12 bone marrow stromal cells in the presence of soluble RANKL, 1,25(OH)D3 and PGE2	↓ TRAP + cells induced by 1,25(OH)D3 by MK-4 (5 × 10^−6^ M)
		↓ TRAP + cells induced by PGE2 by GGOH (5 × 10^−6^ M)
		GGOH (5 × 10^−6^ M) prevents loss of actin ring induced by alendronate (5 × 10^−5^ M) and risedronate (5 × 10^−5^ M)
van Beek et al. (2006)	Foetal metatarsals labelled with calcium-45	↑ RA receptor mRNA expression by RA (10^−5^ M), GGOH (10^−4^ M), GGA (10^−5^ M)
		GGOH (10^−4^ M) ↑ osteoclastic resorption suppressed by ibandronate
Fisher et al. (2013)	Osteoclasts derived from long bones of rabbit and cultured on bovine bone slides for 3 days with BPs with/without GGOH	GGOH (1 × 10^−5^ M) ↑ osteoclastic resorption suppressed by alendronate, ibandronate, zoledronate at low doses but not high doses
Ohgi et al. (2013)	Murine bone marrow cells induced with RANKL + MCSF, and RAW264.7 macrophages induced with RANKL, treated with BP ± GGOH.	↑ outward and inward Cl^−^ current suppressed by zoledronate in an acidic environment by GGOH (3 × 10^−5^ M)
Nakagawa et al. (2015)	Osteoclast precursors from ICR mice treated with/without RANKL/zoledronate/GGOH for 2 days	GGOH (1 × 10^−4^ M) ↑ multinucleated cells and NFATc1 and carbonyl anhydrase II expression suppressed by zoledronate
Fliefel et al. (2019)	Commercialised human osteoclasts treated with zoledronate (1 × 10^−7^, 2.5 × 10^−5^, 1 × 10^−4^ M) and GGOH (1 × 10^−5^–8 × 10^−5^ M) for 7 days	GGOH alone ↑ cell viability at low doses (1 × 10^−5^–4 × 10^−5^ M), ↓ it at high dose (8 × 10^−5^ M) with or without zoledronate
		GGOH at 1 × 10^−5^ M ↑ TRAP + cells but ↑ apoptosis at 8 × 10^−5^ M
		GGOH ↑ Rap1A/B protein expression suppressed by zoledronate
Zafar et al. (2020)	Human peripheral mononuclear cells from three healthy women were cultured with RANKL and MCSF, and with the zoledronate and GGOH treatment for 2 days	GGOH (5 × 10^−5^ M) preserves osteoclasts morphology exposed to zoledronate
		GGOH (5 × 10^−5^ M) ↓ TNF, CXCL9, CXCL10 expression upregulated by zoledronate alone

Abbreviation↓, decrease; ↑, increase; +, positive; 1,25(OH)D, 1,25-dihydroxyvitamin D3; BP, bisphosphonates; CXCL9, chemokine ligand 9; CXCL10, chemokine ligand 10; DC-STAMP, dendrocyte expressed seven transmembrane protein; GGOH, geranylgeraniol; MCSF, macrophage colony-stimulating factor; MK-4, menatetrenone; RANKL, receptor activator of nuclear factor kappa-Β ligand; NFATc1, nuclear factor of activated T-cells, cytoplasmic 1; TRAP, tartrate-resistant acid phosphatase.

### Effects of GGOH Alone on Osteoblasts

Evidence on the effects of GGOH on osteoblasts is limited compared to its effects on osteoclasts. Still et al. reported that GGOH alone can increase the colony-forming unit in rat bone marrow culture (Still et al., 2003). However, GGOH at a high dose (80 μM) is toxic to human mesenchymal stem cells which serve as osteoblast precursors (Fliefel et al., 2020). GGOH at low doses (10–40 μM) promoted human osteoblast viability but reduced it at a high dose (80 μM) (Fliefel et al., 2019). GGOH does not promote osteoblast migration (Hagelauer et al., 2015). Patntirapong et al. reported that GGOH alone does not alter the viability of MC3T3-E1 murine pre-osteoblasts but increases mineral deposition in culture (Patntirapong et al., 2021) (Table 3).

**TABLE 3:** The effects of GGOH on osteoblasts.

GGOH alone		
**Authors (years)**	**Study design**	**Major findings**
Yoshida et al. (2009)	MC3T3-E1 pre-osteoblastic cells were cultured with GGOH with geranylgeranyl transferase I inhibitor in serum-deprived media	GGOH (10^−5^ M) ↓ caspase-3 activity and cell death induced by geranylgeranyl transferase I inhibitor but not Rho kinase inhibitor
Hagelauer et al. (2015)	Commercialised human osteogenic cells treated with zoledronate, GGOH, farnesol or other isoprenoids	GGOH alone: High dose (5 × 10^−5^–1 × 10^−4^ M) ↓ cell viability, no effects on migration (10^−5^–10^−4^ M)
**GGOH in the presence of BPs**		
Still et al. (2003)	Bone marrow cells from Wistar rats were harvested and incubated with various bisphosphonates and with or without GGOH.	GGOH (10^−3^ M) ↑ colony formation in culture exposed/unexposed to suppressed by alendronate (10^−5^ M) and risedronate (10^−5^ M)
Ziebert et al. (2011)	Commercially available human osteogenic cells cultured with BPs with or without GGOH for 72 h. All BP at 5 × 10^−5^ M, GGOH at ×10^−5^ M	GGOH ↑ the viability suppressed by ibandronate, pamidronate, zoledronate
		GGOH ↑ cell migration suppressed by zoledronate
		GGOH reversed cytoskeletal distortion in all cells caused by ibandronate, pamidronate, zoledronate
Hagelauer et al. (2015)	Commercialised human osteogenic cells treated with zoledronate, GGOH, farnesol or other isoprenoids	GGOH alone: High dose (5 × 10^−5^–1 × 10^−4^ M) ↓ cell viability, no effects on migration (10^−5^–10^−4^ M)
		GGOH + zoledronate: ↑ cell viability and migration suppressed by zoledronate acid at all concentrations (10^−5^–10^−4^ M)
		GGOH reverses destructive cellular morphology induced by zoledronate
		Other isoprenoids including farnesol show no similar antagonism
Zafar et al. (2016)	Human osteoblasts from the mandibular alveolar bone of three healthy women were cultured with zoledronate and geranylgeraniol for 72 h	GGOH (5 × 10^−5^ M) ↑ cell viability, migration, nodule formation suppressed by zoledronate
		GGOH preserves cell morphology in cells exposed to zoledronate
Fliefel et al. (2019)	Commercialised human osteoblasts treated with zoledronate (1 × 10^−7^, 2.5 × 10^−5^, 1 × 10^−4^ M) and GGOH (1 × 10^−5^–8 × 10^−5^ M) for 7 days	GGOH alone ↑ cell viability at low doses (1 × 10^−5^–4 × 10^−5^ M), ↓ it at high dose (8 × 10^−5^ M) with or without zoledronate
		GGOH ↑ Rap1A/B protein expression suppressed by zoledronate
Fliefel et al. (2020)	Commercialised human mesenchymal stem cells were treated with zoledronate, GGOH or both for 7 days	At high dose (8 × 10^−5^ M), GGOH alone or with zoledronate at high dose ↓ cell viability
		At low dose (1 × 10^−5^–4 × 10^−5^ M), GGOH ↑ cell viability suppressed by zoledronate
Mungpayabarn and Patntirapong (2021)	MC3T3 cells were treated with alendronate (10^−5^ M) and GGOH (5 × 10^−5^ M)	GGOH added at early time points ↑ mineralisation suppressed by alendronate
		GGOH preserves cellular morphology in cells exposed to GGOH
		GGOH ↑ FGF2, VEGF, VEGFR2, COL1, OPN expressions in cells exposed to alendronate. OPN and VEGF upregulations are sustained for up to 7 days
Patntirapong et al. (2021)	MC3T3-E1 was treated with alendronate (5 × 10^−6^, 1 × 10^−5^, 5 × 10^−5^ M) and GGOH (1 × 10^−5^, 5 × 10^−5^ M)	GGOH alone did not affect cell viability but ↑ viability and ↓ apoptosis in cells exposed to alendronate
		GGOH relieves G2/M phase accumulation due to alendronate
		GGOH preserves actin stress fibre and destructive cell morphology caused by alendronate
		GGOH alone or with alendronate ↑ mineralisation
Singhatanadgit et al. (2021)	Commercialised human mesenchymal stem cells were cultured with zoledronate (0–5 × 10^−5^ M) with or without GGOH (0–10^−4^ M)	GGOH ↑ viability, mineralisation and ↓ cell cycle arrest through reactivation of Rho and YAP in mesenchymal stem cells exposed to zoledronate

Abbreviation↓, decrease; ↑, increase; COL1, type I collagen; GGOH, geranylgeraniol; FGF2, fibroblast growth factor-2; OPN, osteopontin; VEGF, vascular endothelial growth factor; VEGFR2, vascular endothelial growth factor receptor 2.

### Effects of GGOH on Osteoblasts in the Presence of Bisphosphonates

Yoshida et al. demonstrated the ability of GGOH to prevent activation of caspase-3 and apoptosis by a geranylgeranyl transferase I inhibitor, probably by increasing the substrate pool for prenylation of GTPase (Yoshida et al., 2009). This response of GGOH could be extended to its reversal of the negative effects bisphosphonate have on osteoblasts or their precursors. Bisphosphonate treatment dramatically decreased viable mesenchymal stem cells by inhibiting geranylgeranylation. GGOH preserved these important differentiator cells by reversing apoptosis and cell cycle arrest through Rho-dependent yes-associated protein activation pathway (Singhatanadgit et al., 2021). Suppression of colony formation in rat bone marrow culture by alendronate and risedronate can be reversed by GGOH (Still et al., 2003). By extension, GGOH could reverse the negative effects of bisphosphonates on osteoblasts. In fact, multiple studies have reported that GGOH reverses bisphosphonate-mediated reductions in osteoblast viability, migration and mineralisation (Ziebart et al., 2011; Hagelauer et al., 2015; Patntirapong et al., 2021). Of note, zoledronate causes apoptotic features in human osteoblasts from the mandibular alveolar bone, such as chromatin condensation, organelle disintegration and irregular-shaped cellular membrane, which are reversed by GGOH (Zafar et al., 2016). GGOH also reverses destructive cellular morphology caused by alendronate, such as reduction in actin stress fibres, cell debris and condensation of nuclei in MC3T3-E1 cells (Patntirapong et al., 2021). Of note, actin cytoskeletal reorganisation is critical for osteoblast differentiation and functions (Suzuki et al., 2019). GGOH also relieves cell cycle arrest of MC3T3-E1 caused by alendronate (Patntirapong et al., 2021).

The rescue action of GGOH on osteoblast dysfunction could be attributed to the upregulation of Rap1A/B protein suppressed by zoledronic acid (Fliefel et al., 2019). This event eventually leads to the correction of the expression of genes coding for bone formation protein, such as fibroblast growth factor-2, vascular endothelial growth factor (VEGF), VEGF receptor 2 (VEGFR2), collagen 1 and osteopontin. Of note, VEGF and VEGFR2 are modulators of bone healing by promoting osteoblast differentiation and maturation, angiogenesis and recruitment of immune cells (Grosso et al., 2017; Hu and Olsen, 2017). Zafar et al. reported that GGOH suppressed zoledronate-initiated upregulation of 4 genes in primary human osteoblasts coding for regulators of angiogenesis, such as platelet-derived growth factor-beta polypeptide, chemokine (C-C motif) ligand 2, CD55 and thrombospondin 1 (Zafar et al., 2016). However, GGOH did not normalise the expression of osteogenic genes affected by zoledronate (Zafar et al., 2016). This finding indicates that the rescue effects of GGOH on osteoclasts could be more prominent than on osteoblasts.

Overall, GGOH alone promotes osteoblast viability at a low dose but may be cytotoxic to the cells at a high dose. At a low dose, it can prevent bisphosphonate-induced osteoblast damage (Table 3).

### Effects of GGOH on Other Cells

Cozin et al. found that GGOH alone is inert to the primary gingival fibroblasts, but it reverses the suppression of proliferation, migration and adhesion defect due to loss of cell-substratum adhesion and F-actin bundles caused by zoledronate (Cozin et al., 2011). However, GGOH could not reverse the negative effects of pamidronate in the same study due to the more extensive damage marked by activation of caspase-3 and apoptosis (Cozin et al., 2011). Similar findings were obtained by Ziebart et al. and Hagelauer et al. (Ziebart et al., 2011; Hagelauer et al., 2015). Ziebart et al. also showed that GGOH could only rescue the viability of human gingival fibroblasts suppressed by zoledronate, but not ibandronate and pamidronate (Ziebart et al., 2011). However, GGOH restores migration suppressed by all nitrogen-containing bisphosphonates tested (Ziebart et al., 2011). Zafar et al. reported that GGOH alone reduces the viability of human gingival fibroblasts in contrast to the promoting effects of farnesol. GGOH reverses the degenerative changes, upregulation of bone morphogenetic protein-2 and downregulation of VEGF-A induced by zoledronic acid (Zafar et al., 2014).

The effects of GGOH on human umbilical vein endothelial cells (HUVECs) have also been examined. GGOH alone at high doses (50–100 µM) is toxic to HUVEC and does not affect cell migration (Hagelauer et al., 2015). On the other hand, GGOH antagonises the effects of zoledronate in suppressing HUVEC viability and migration (Hagelauer et al., 2015). Similar effects were observed with ibandronate but not with pamidronate (Ziebart et al., 2011).

Overall, GGOH prevents nitrogen-containing bisphosphonate-mediated damage in gingival fibroblasts and HUVECs, which can help oral mucosal healing and angiogenesis (Table 4).

**TABLE 4:** The effects of GGOH on other cells.

Authors (years)	Study design	Major findings
Cozin et al. (2011)	Primary gingival fibroblasts were exposed to zoledronate or pamidronate with or without GGOH.	GGOH alone is inert to the cells
		GGOH ↑ cell proliferation and migration, ↓ adhesion defect (cell-substratum adhesion and F-actin bundles) caused by zoledronate effectively (3 × 10^−5^ M) and not pamidronate (6 × 10^−5^ M)
Ziebart et al. (2011)	Commercially available HUVEC and human gingival fibroblasts cultured with BPs with or without GGOH for 72 h. All BPs at 5 × 10^−5^ M, GGOH at 10^−5^ M	HUVEC: GGOH ↑ cell viability and migration suppressed by ibandronate and zoledronate
		Fibroblasts: GGOH ↑ cell viability suppressed by zoledronate; ↑ cell migration by ibandronate, pamidronate and zoledronate
		GGOH cellular cytoskeletal distortion in cells exposed to all BPs
Zafar et al. (2014)	Human gingival fibroblasts from 5 female patients were cultured with 3 × 10^−5^ M zoledronate with or without farnesol (10^−5^ M) or GGOH (5 × 10^−5^ M) for 72 h	GGOH alone ↓ cell viability but ↑ cell viability in the presence of zoledronate
		GGOH preserves the morphology of cells exposed to zoledronate
		GGOH ↓ BMP2 and VEGF-A expression induced by zoledronate
Hagelauer et al. (2015)	Commercialised human gingival fibroblasts and HUVEC were treated with zoledronate, GGOH, farnesol or other isoprenoids	GGOH alone: High dose (5 × 10^−5^–10^−4^ M) ↓ cell viability, no effects on migration (10^−5^–10^−6^ M)
		GGOH + zoledronate: ↑ cell viability and migration suppressed by zoledronate at all concentrations (10^−5^–10^−6^ M)
		GGOH reverses destructive cellular morphology

Abbreviation↓, decrease; ↑, increase; BMP-2, bone morphogenetic protein-2; BP, bisphosphonate; HUVEC, human umbilical vein endothelial cells; GGOH, geranylgeraniol; VEGF-A, vascular endothelial growth factor-A.

### Effects of GGOH on BRONJ *in vivo*

Only three animal studies investigating the effects of GGOH supplementation in animal models of BRONJ were identified in our literature search. Koneski et al. treated male Wistar rats with zoledronate (0.06 mg/kg) for 5 weeks and extracted their first mandibular molar. GGOH solution was administered at the tooth socket at 5 mM during the last 2 weeks of the experiment (Koneski et al., 2018). GGOH treatment attenuates soft tissue inflammation and jawbone defect in the rats (Koneski et al., 2018). As evidence, 80% of the untreated rats show signs of osteonecrosis while two-thirds of the treated rats show improved vascularity, tissue granulation, osteoblast lining and epithelial coverage, as well as reduced empty lacunae (Koneski et al., 2018).

Nagaoka et al. treated male C57BL/6J mice (6-week-old) with zoledronate (250 μg/kg, i. p.) and lipopolysaccharide (250 μg/kg, i. p.) twice a week (Nagaoka et al., 2015). The first maxillary molar was extracted after 2 weeks, and mice were treated with GGOH or geranylgeranyl pyrophosphate (GGPP) for 4 weeks after the tooth extraction. The researchers showed that GGOH and GGPP reverse the reduction of bone mineral density, bone volume and TRAP-positive cells at the tooth socket caused by zoledronate and lipopolysaccharide (Nagaoka et al., 2015).

In the study of Chen et al., male C57BL/6J mice (4-week-old) were given zoledronate (250 μg/kg, i. p.) twice a week and their first maxillary molar were extracted 1 week after zoledronate treatment. GGOH (250 μg/kg) was administered together with zoledronate after tooth extraction for 4 weeks (Chen et al., 2021). Zoledronate stimulates osteocyte and pre-osteoblast apoptosis in mice and suppressed efferocytosis of macrophages on apoptotic cells (Chen et al., 2021). GGOH reverse the negative effects of zoledronate on efferocytosis by restoring Rac1 homeostasis and shuttling Rac1 at the cellular membrane. Consequently, GGOH reduces osteocytic apoptosis at the alveolar crest and improves bone healing of the tooth sockets in mice (Chen et al., 2021). This is the only study that investigates the protective effects of GGOH on osteocytes under the influence of bisphosphonates. Considering the role of osteocytes as a master regulator of bone remodelling, more research should be invested to understand the effects of GGOH on osteocytes.

Overall, both topical and systemic administrations of GGOH are proven to prevent BRONJ in animal models (Table 5). Some limitations should be noted in all three animal studies, wherein small animals were used as a model. Significant intracortical bone remodelling important for cortical bone in humans is absent in rodents (Jilka, 2013). Therefore, larger animals such as minipigs, rabbits, dogs or sheep could be a better model of BRONJ because they have intracortical remodelling, barring their high cost (Allen, 2015). All studies also used male animals as a model, probably to minimise the influence of hormonal variation on bone in female animals. However, due to the prevalent use of bisphosphate among postmenopausal women to treat osteoporosis (Bor et al., 2015), women would be receiving the most benefits from GGOH intervention. Thus, the efficacy of GGOH in reversing BRONJ should be examined in animal models with oestrogen deficiency. However, sex difference in the occurrence of BRONJ is contentious, and women do not seem to be more susceptible than men (Kharazmi et al., 2014). Overall, more *in vivo* studies with longer-term applications are needed to show the efficacy of GGOH in managing BRONJ.

**TABLE 5:** The effects of GGOH *in vivo* model of BRONJ.

Authors (years)	Study design	Major findings
Nagaoka et al. (2015)	Model: Male C57BL/6J mice of 6 weeks were injected with zoledronate (250 μg/kg) and LPS (250 μg/kg) (i.p.) twice a week. First maxillary molar was extracted after 2 weeks.	GGOH and GGPP ↑ bone mineral density, bone volume and TRAP + cells at the tooth-extracted socket caused by zoledronate + LPS.
	Treatment: GGOH (250 μg/kg) or GGPP (250 μg/kg) were injected together with zoledronate and LPS after tooth extraction for 4 weeks	
Koneski et al. (2018)	Model: Male Wistar rats were given zoledronate (0.06 mg/kg) for 5 weeks and underwent first mandibular molar extraction	GGOH ↓ inflammation of soft tissue and bone defect
	Treatment: GGOH solution at the extraction socket at 5 mM for the last 2 weeks	Less rats treated with GGOH shows osteonecrosis in tissue adjacent to the extraction
		GGOH improves vascularity, tissue granulation, osteoblast lining and epithelial coverage and ↓ empty bone lacunae
Chen et al. (2021)	Model: male C57BL/6J mice (4 weeks old) were given zoledronate (250 μg/kg) twice a week (i.p.). One week after zoledronate treatment, the first maxillary molar was extracted	GGOH restores efferocytosis of macrophages on apoptotic cells by upregulating Rac1 homeostasis and expression in the membrane
	Treatment: GGOH (250 μg/kg) were injected twice a week together with zoledronate acid after tooth extraction for 4 weeks	GGOH ↓ osteocytic apoptosis at the alveolar crest and improved bone healing of sockets (new woven bone, periosteum at alveolar crest) in mice

Abbreviation↓, decrease; ↑, increase; i.p., intraperitoneal; GGPP, geranylgeranyl pyrophosphate; GGOH, geranylgeraniol; LPS, lipopolysaccharide.

### Perspective

Several important points should be considered in developing GGOH as an interventional agent for BRONJ. Several authors have proposed topical application of GGOH in contrast to systemic application, given the concerns that it might antagonise the therapeutic effects of bisphosphonates in the management of cancer and osteoporosis. This approach has been proven effective by Koneski et al. in a rat model of BRONJ using a GGOH solution at 5 mM, which can be translated to humans directly (Koneski et al., 2018). We further propose that GGOH can be incorporated into natural and synthetic dental implants or bone grafts to accelerate the healing process. This aspect would require further investigation at the preclinical stage. In a preprint article, GGOH (4 mM) has been incorporated in bone cement and shown the ability to reverse pamidronate-mediated suppression of osteoclast viability and resorption activity *in vitro* (Feldman et al., 2020).

In addition, dosing inconsistency and penetration of GGOH in deep tissue should be solved. Many *in vitro* studies highlighted that high-dose GGOH could be toxic to gingival fibroblasts, bone cells and HUVECs (Hagelauer et al., 2015; Fliefel et al., 2019). Exposure of human fibroblasts, osteoblasts and HUVECs to GGOH at concentrations below 50 μM appears to be safe (Hagelauer et al., 2015; Fliefel et al., 2019). Thus, delivery of GGOH at a precise dose through a slow-releasing strategy like nanomaterials should be adopted to achieve this. Besides, *in vivo* toxicity data of GGOH is scarce. An oral toxicity study reported that high doses of GGOH (725, 1,450, 2,900 mg/kg body weight) for 90 days induce significant pathological changes in the liver and forestomach in rats. The negative effects of the forestomach were considered to be local irritative effects attributed to oral gavage (Preece et al., 2021). However, the doses used are very high and might not be relevant if topical administration is considered. In the same study, the bacterial mutagenicity test and mammalian cell clastogenicity test of GGOH did not reveal any significant findings (Preece et al., 2021).

In a high-fat diet-induced bone loss study, Chung et al. showed that GGOH supplementation (800 mg/kg diet) for 14 weeks improved glucose metabolism and bone microarchitecture and strength in rats (Chung et al., 2021). GGOH also alters the gut microbiota diversity of the rats by increasing *Butyricicoccus pullicaecorum* (butyrate-producing bacteria) and reducing *Dorea longicatena* (glutamate-producing bacteria) in the caecum (Chung et al., 2021). This observation leads to the question of whether topical application of GGOH alters oral microflora and contributes to the prevention of BRONJ since microflora is one of the contributing factors to the disease. The currently limited evidence shows that GGOH possesses inhibitory action on *Mycobacterium tuberculosis* and *Staphylococcus aureus in vitro*. Its actions on other bacteria are largely unknown (Inoue et al., 2005; Vik et al., 2007).

Lastly, the effects of GGOH could only antagonise the side effects of nitrogen-containing bisphosphonates, not non-nitrogen-containing bisphosphonates, denosumab and antiangiogenic agents (Reszka et al., 1999; van beek et al., 1999). This limitation is due to its specificity in targeting the mevalonate pathway. GGOH intervention might not be effective when the dose of bisphosphonates is high, as in monthly or yearly regimens (Fisher et al., 2013). Recent studies have highlighted that bisphosphonates like zoledronate affect multiple cellular processes, and exert differential effects on cells. For example, stimulation of transient receptor potential cation channel subfamily V member 1 (TRPV1) by zoledronate at nanomolar concentrations increases osteoblast proliferation and mineralisation. This effect is absent in osteoclasts because they do not express TRPV1. However, at micromolar concentration, zoledronate inhibits farnesyl pyrophosphate synthase and geranylgeranyl pyrophosphate, leading to anti-proliferative effects on bone cells (Scala et al., 2019). Zoledronate also binds with KIR6.1/6.2 and the SUR2A/B subunits on ATP-sensitive potassium (KATP) channels of osteoblasts and muscle fibres, which potentially contribute to its musculoskeletal side effects (Maqoud et al., 2021). The effects of GGOH on these processes are not known and should be investigated.

These factors should be considered in applying GGOH in the management of MRONJ clinically in the future.

## Conclusion

BRONJ is a multifactorial disease involving bone trauma, impaired bone healing due to suppressed bone remodelling and angiogenesis, soft tissue toxicity, microbial infection and inflammation of the injured site. The existing evidence shows that GGOH can antagonise the effects of nitrogen-containing bisphosphonates on bone remodelling, resolve soft tissue toxicity and promote angiogenetic factors locally. These properties make GGOH a potential adjunctive agent in the management of BRONJ. Further investigation on the method of delivery, precise dosing, and mechanisms of action should be undertaken both pre-clinically and clinically. With GGOH being generally recognized as safe and widely available as a dietary supplement, the nutrient can now be validated in well-planned clinical trials to show its efficacy in mitigating BRONJ in humans.
